# Full‐Arch Trans‐Sinus Implant Placement Using a Stackable Surgical Guide With Digitally Planned Antrostomy: A Case Report

**DOI:** 10.1155/crid/2384584

**Published:** 2026-04-15

**Authors:** Mattia Manfredini, Matteo Pellegrini, Pier Paolo Poli, Carlo Maiorana, Franco Sanseverino, Mario Beretta

**Affiliations:** ^1^ University of Milan, Department of Biomedical, Surgical and Dental Sciences, Via della Commenda 10, 20122, Milan, Italy, unimi.it; ^2^ Fondazione IRCCS Ca’ Granda Ospedale Maggiore Policlinico, Maxillo-Facial Surgery and Dental Unit, 20122, Milan, Italy, policlinico.mi.it; ^3^ Consultant, Milan, Italy

**Keywords:** digital implant planning, maxillary full-arch rehabilitation, stackable surgical guide, trans-sinus implant

## Abstract

**Introduction:**

This report presents a fully digital workflow for maxillary full‐arch rehabilitation that integrates trans‐sinus implant placement into a stackable guided surgery system. The technique incorporates a digitally planned antrostomy guide to enable controlled lateral sinus access.

**Methods:**

A sequential stackable guide protocol was developed, including key, base, drive, and prosthetic templates, together with a custom antrostomy guide. The system was designed to translate the virtual plan into precise implant positioning and reproducible lateral window preparation for trans‐sinus implant placement.

**Results:**

The digital workflow enabled accurate localization of the sinus window and guided insertion of a tilted trans‐sinus implant along the planned trajectory. The combined guide system provided controlled sinus access and stable implant placement suitable for immediate loading.

**Conclusion:**

Integrating a digitally planned antrostomy guide within a stackable surgical system enhances precision in full‐arch rehabilitations requiring trans‐sinus implants. This digital technique expands the applicability of guided surgery to challenging posterior maxillary scenarios.

## 1. Introduction

Stackable guides have become widely adopted in full‐arch implant rehabilitations, offering improved surgical precision and workflow efficiency. Multiple studies have confirmed their accuracy, despite variations in design and execution [1–6]. These systems enable the sequential use of diagnostic, surgical, and prosthetic templates based on a shared reference, ensuring precise control from bone reduction to immediate loading [1–6]. Compared with conventional single‐piece guides, stackable systems allow greater flexibility, improved intraoperative stability, verification at each surgical step, and more predictable management of complex full‐arch workflows, particularly in immediate‐loading protocols [4, 5, 7–10].

In parallel, the trans‐sinus implant approach has emerged as a viable solution for atrophic posterior maxillae, allowing long implants to traverse the sinus and engage distant cortical anchorage [11–17]. Traditional lateral window sinus lift procedures, although effective, are associated with several limitations, including increased surgical morbidity, prolonged healing time before implant loading, and a nonnegligible risk of Schneiderian membrane perforation. These factors make the trans‐sinus approach an attractive alternative in selected cases requiring immediate stability and reduced overall treatment time [18–20]. While guided lateral sinus lift procedures have been proposed, current literature is limited to grafting protocols without implant placement through the sinus [21, 22].

To address the need for a reproducible and accurate lateral antrostomy aligned with the virtual implant path, a dedicated antrostomy guide was integrated into the stackable workflow. This tool facilitates precise translation of the digital plan into clinical execution, particularly in challenging anatomical scenarios.

## 2. Case Report

### 2.1. Diagnosis and Etiology

A 65‐year‐old patient was referred to the Maxillo‐Facial Surgery and Dental Unit at Fondazione IRCCS Ca’ Granda Ospedale Maggiore Policlinico in Milan, Italy, for full‐arch maxillary rehabilitation. The patient’s medical history was significant for atrial fibrillation managed with long‐term anticoagulation therapy (warfarin, daily regimen). He denied tobacco use.

Clinically, the patient presented with a completely edentulous maxillary arch (Figures 1A, B). The patient reported that extraction of the remaining maxillary teeth had been performed 4 months prior to the first visit, due to terminal dentition with poor periodontal and restorative prognosis. The edentulous ridge exhibited regular postextraction healing and provided a favorable foundation for full‐arch prosthetic rehabilitation.

Figure 1Pretreatment intraoral views of the edentulous maxillary arch, showing frontal (A) and palatal (B) perspectives prior to digital planning and full‐arch implant rehabilitation.

**Figure:**
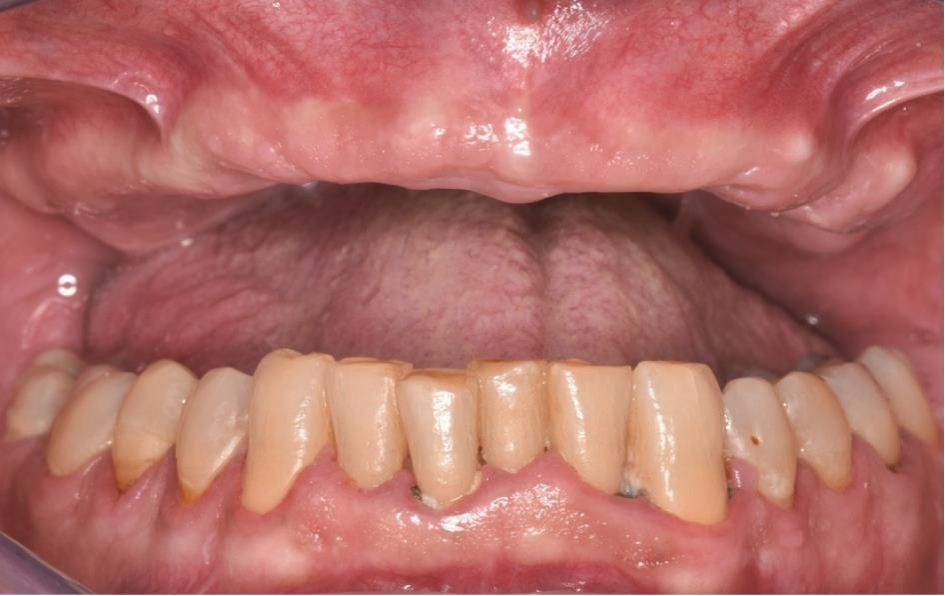
(A)

**Figure:**
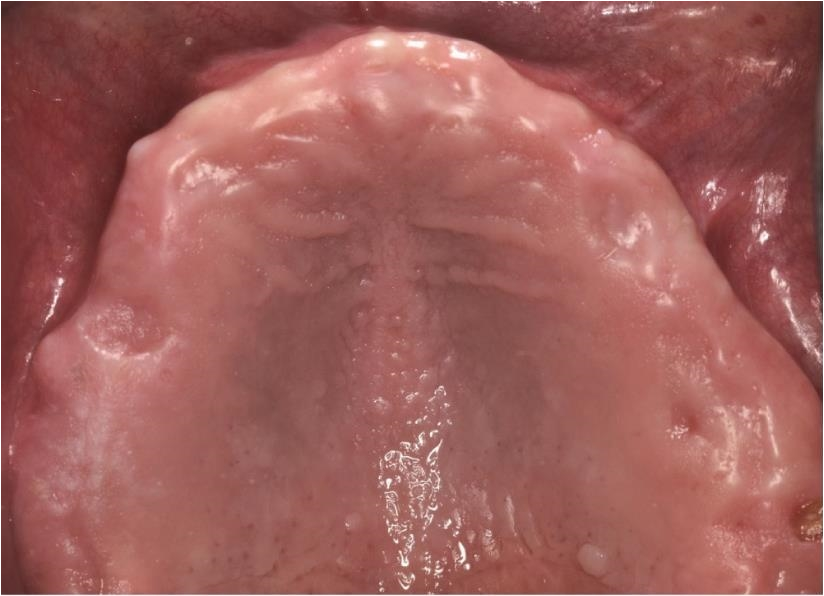
(B)

### 2.2. Treatment Objectives

The aim of the treatment is the full‐arch rehabilitation of the edentulous maxillary arch through a prosthetically driven implant‐supported restoration, ensuring optimal function, esthetics, and long‐term stability. The planned protocol includes the insertion of a tilted trans‐sinus implant to achieve distal cortical anchorage in the posterior maxilla, where severe pneumatization and reduced residual bone height (RBH) contraindicate conventional implant placement. A secondary objective is to perform the rehabilitation using a fully guided, digitally based workflow to minimize surgical invasiveness and improve positional accuracy. The use of a dedicated antrostomy guide is intended to allow controlled lateral sinus access and guided elevation of the Schneiderian membrane, thereby enabling the correct trajectory of the trans‐sinus implant without extensive grafting procedures. Immediate loading with a screw‐retained provisional prosthesis is planned to restore mastication and phonetics while supporting soft tissue conditioning and patient comfort during osseointegration.

### 2.3. Treatment Alternatives

The first possible alternative is a conventional sinus augmentation procedure followed by placement of standard‐length implants, requiring delayed loading and multiple surgical phases. This approach allows restoration of posterior bone volume but may increase morbidity, surgical time, and patient discomfort due to grafting procedures. Another alternative is a full‐arch rehabilitation using a conventional All‐on‐Four configuration without trans‐sinus implant placement, positioning all implants in the anterior maxilla. While this avoids sinus manipulation, the reduced posterior support may limit distal extension, increase cantilever length, and compromise biomechanical distribution under occlusal load. A further option is the placement of short or ultra‐short implants in the posterior maxilla without sinus elevation. This approach reduces surgical invasiveness but may not provide sufficient cortical anchorage in cases of severe sinus pneumatization, compromising long‐term prosthetic stability. The last alternative is a removable complete denture, which restores function and esthetics with minimal surgical intervention. However, this solution offers reduced retention, stability, and patient comfort compared to fixed implant‐supported rehabilitation, particularly in cases of advanced maxillary atrophy.

### 2.4. Treatment Progress

Informed consent was obtained from the patient prior to initiating the treatment plan. A full clinical and radiographic evaluation confirmed severe posterior maxillary atrophy consistent with sinus pneumatization, whereas anterior bone volume was sufficient for implant anchorage. Preliminary impressions were obtained using alginate material (Hydrogum 5; Zhermack), followed by definitive maxillary and mandibular impressions with a polyvinyl siloxane material (Elite HD+; Zhermack). Maxillomandibular records were taken with wax rims, and a complete denture was fabricated and delivered post‐extraction to restore function and serve as an esthetic reference during healing. The prosthesis was initially relined with a soft tissue conditioner (Soft‐Liner; GC Europe) and subsequently with a long‐term reline material after 2 months (Soft Reline GC; GC Europe) to ensure stability prior to digital planning.

After complete soft tissue healing, the edentulous maxilla, the relined prosthesis, the opposing arch, and the occlusion were scanned using an intraoral scanner (TRIOS 5; 3Shape) and exported as STL files. The datasets were imported into CAD software (Exocad; exocad GmbH) for alignment and diagnostic virtual waxing. A prototype was then 3D‐printed in biocompatible clear resin (MED610; Stratasys) using a multimaterial 3D printer (J5 DentaJet; Stratasys). Asymmetric radiopaque markers were incorporated into the intaglio surface to enable radiographic matching. With the prototype positioned intraorally, a cone‐beam computed tomography (CBCT) scan was performed (GiANO HR; NewTom), and STL and DICOM files were aligned using dedicated planning software (RealGUIDE; 3DIEMME) based on marker matching (Figure 2A–D).

Figure 2Virtual planning of a full‐arch rehabilitation using a digitally guided stackable system, showing a tilted trans‐sinus implant directed beyond the nasal notch to engage highly corticalized bone at the level of the lateral wall of the maxillary sinus in site 1.5 (A), straight implants placed according to a prosthetically driven trajectory in sites 1.2 (B) and 2.2 (C), and a tilted implant in site 2.5 to maximize anterior–posterior spread (D).

**Figure:**
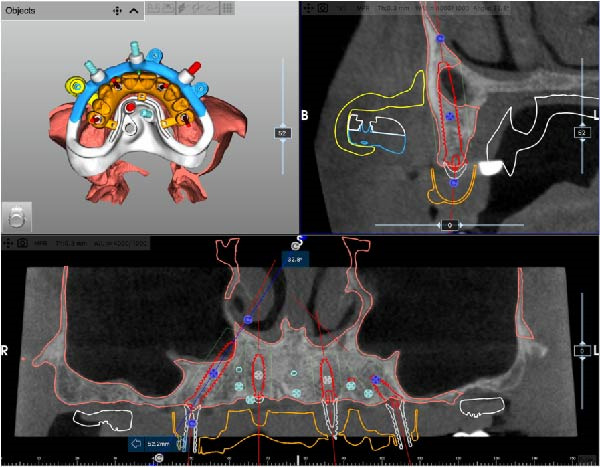
(A)

**Figure:**
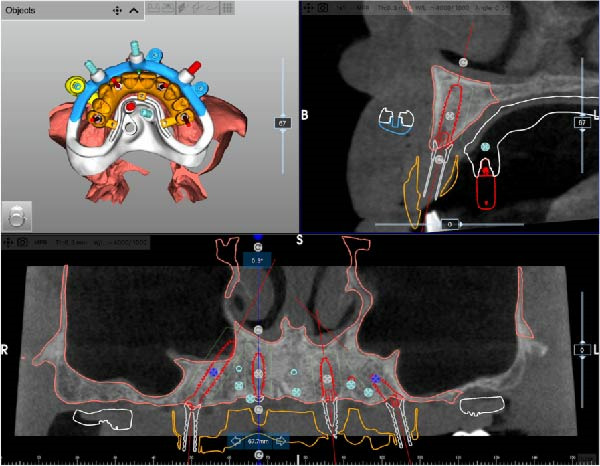
(B)

**Figure:**
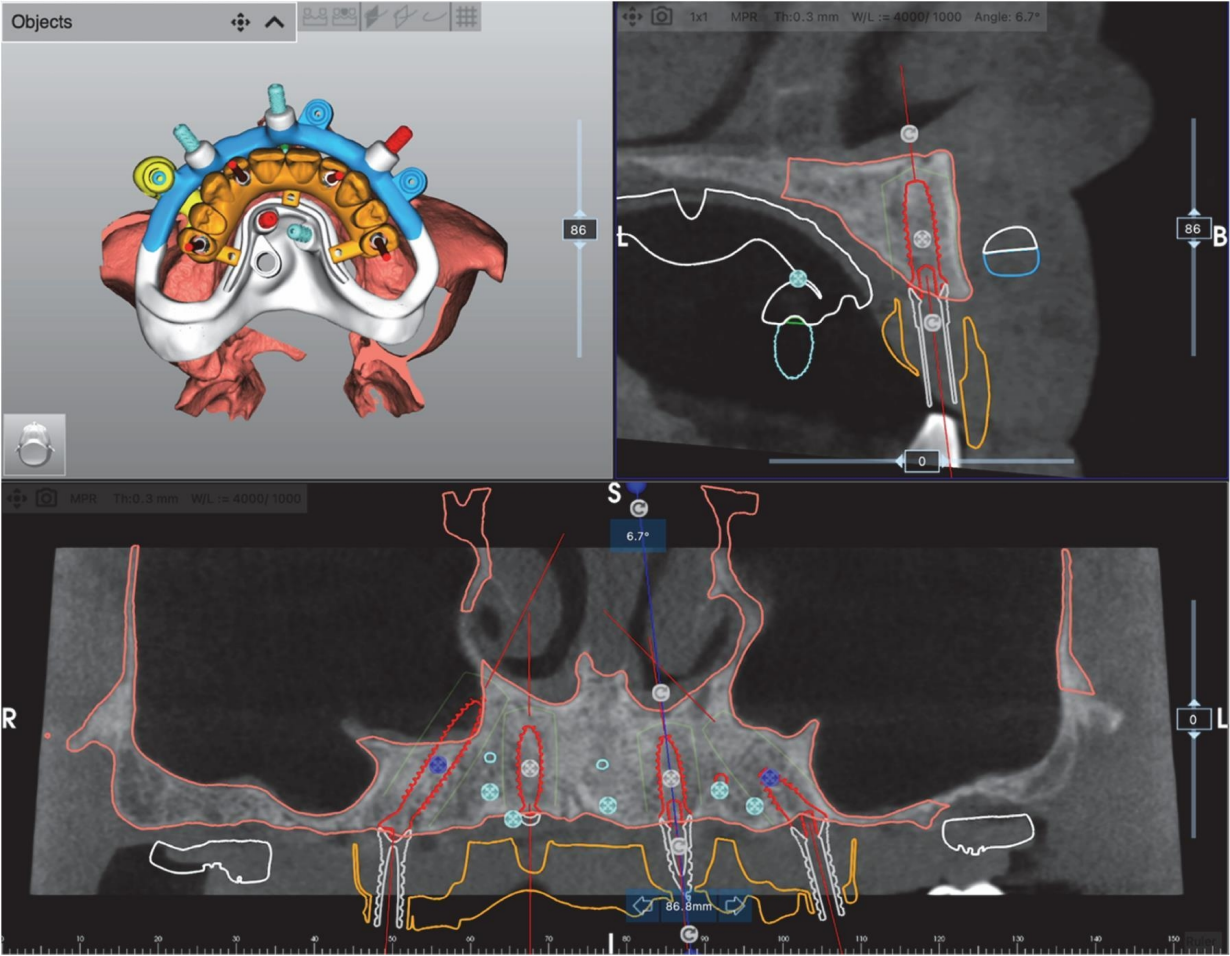
(C)

**Figure:**
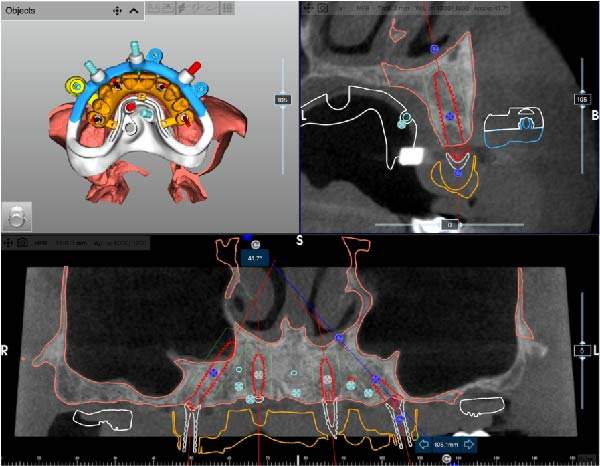
(D)

Implant positions were virtually planned following a prosthetically driven full‐arch approach, including a tilted trans‐sinus implant directed beyond the nasal notch to engage highly corticalized bone at the level of the lateral wall of the maxillary sinus in order to obtain stable distal anchorage. A complete stackable guide system (PCube; Oxy Implant Dental System) was digitally designed, consisting of a key template, a titanium‐reinforced base template, a milled titanium drive template, a resin antrostomy guide, and a prosthetic template for immediate loading (Figure 3A–G).

Figure 3Digitally planned stackable surgical system showing the key template replicating the provisional prosthesis for occlusal positioning and fixation to the base template (A), the milled titanium drive template guiding drilling and implant insertion (B), axial, sagittal, and coronal views of the resin antrostomy guide defining the lateral sinus window (C–E), the prosthetic template designed for immediate loading (F), and the final screw‐retained provisional prosthesis replicating the occlusal morphology for passive fit (G).

**Figure:**
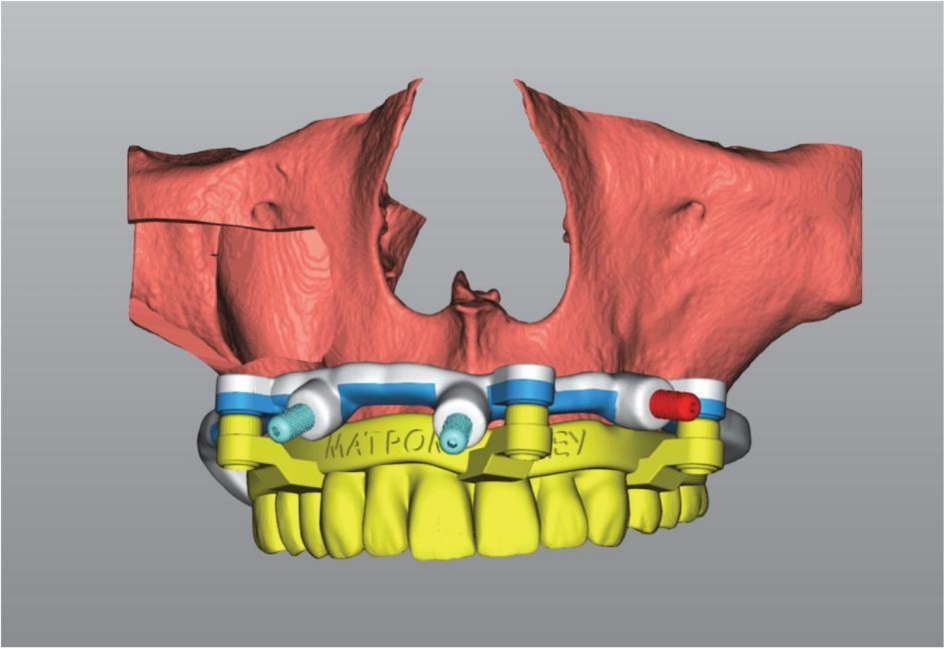
(A)

**Figure:**
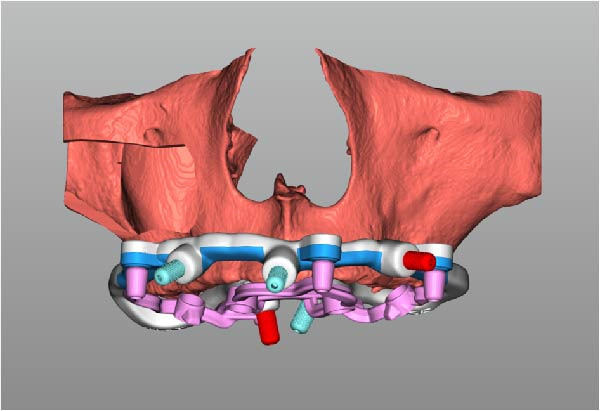
(B)

**Figure:**
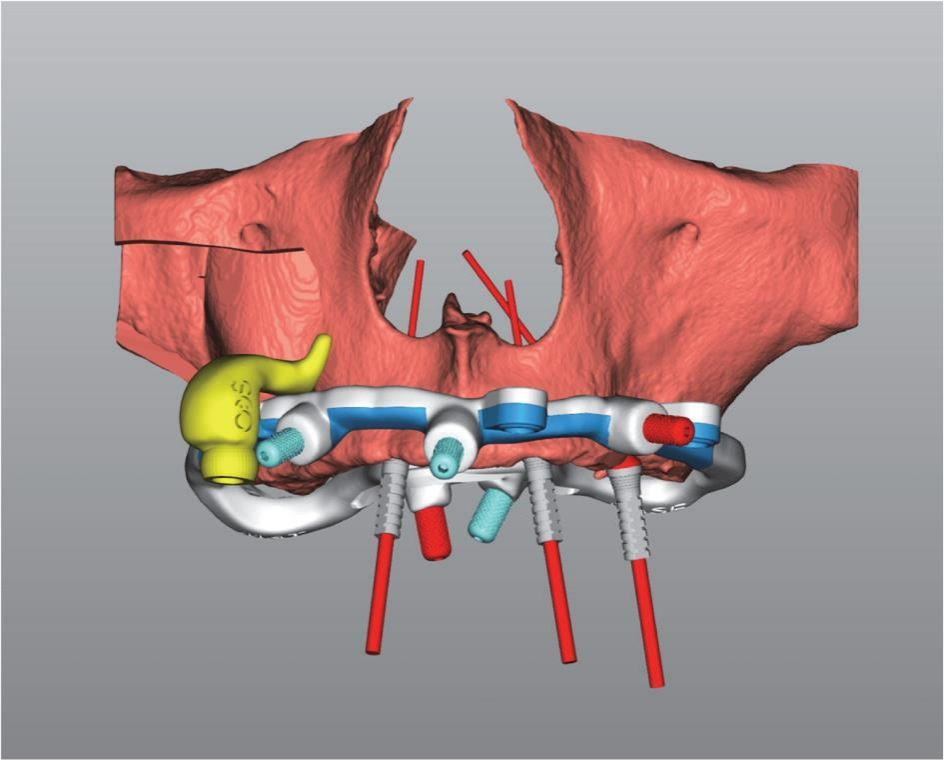
(C)

**Figure:**
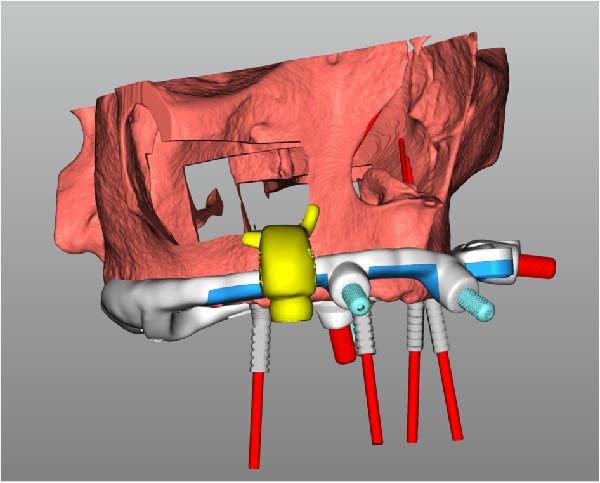
(D)

**Figure:**
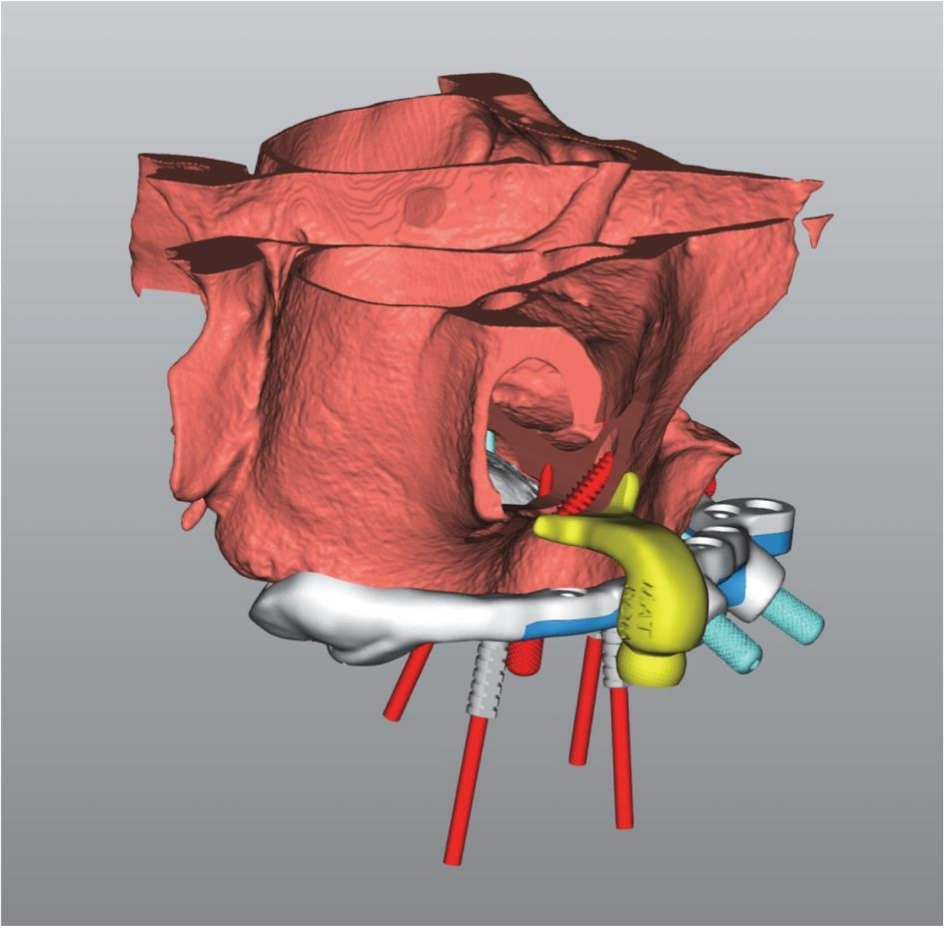
(E)

**Figure:**
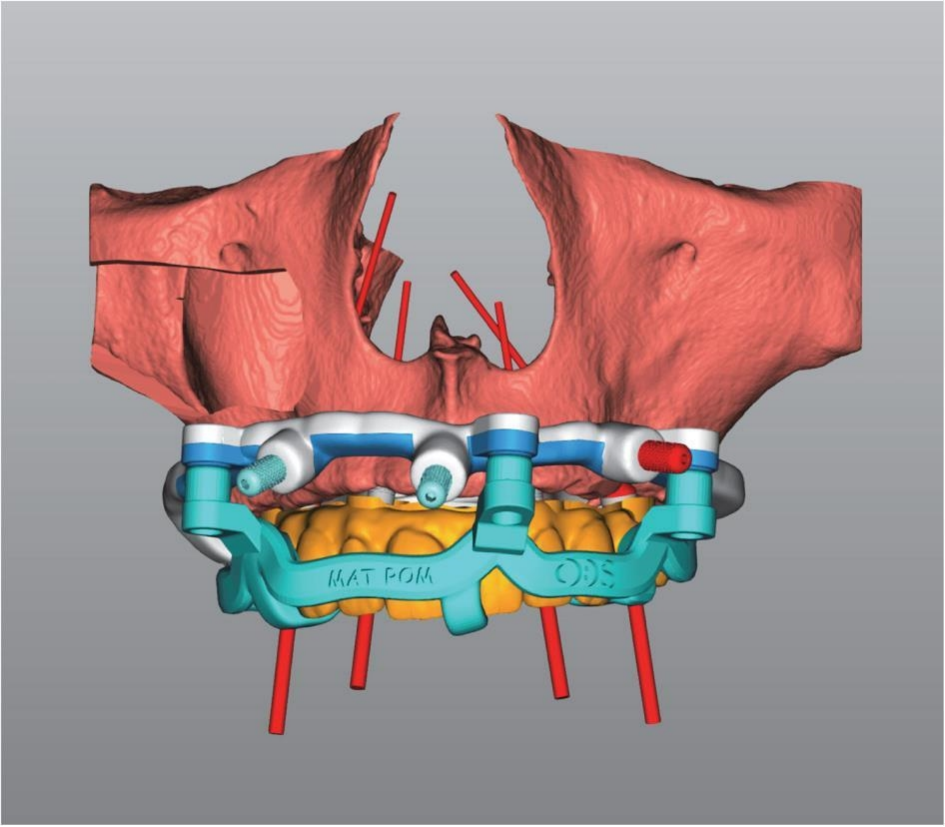
(F)

**Figure:**
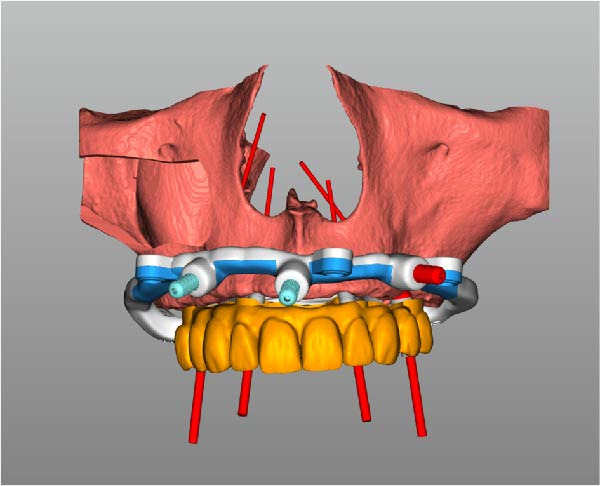
(G)

**Figure 5:**
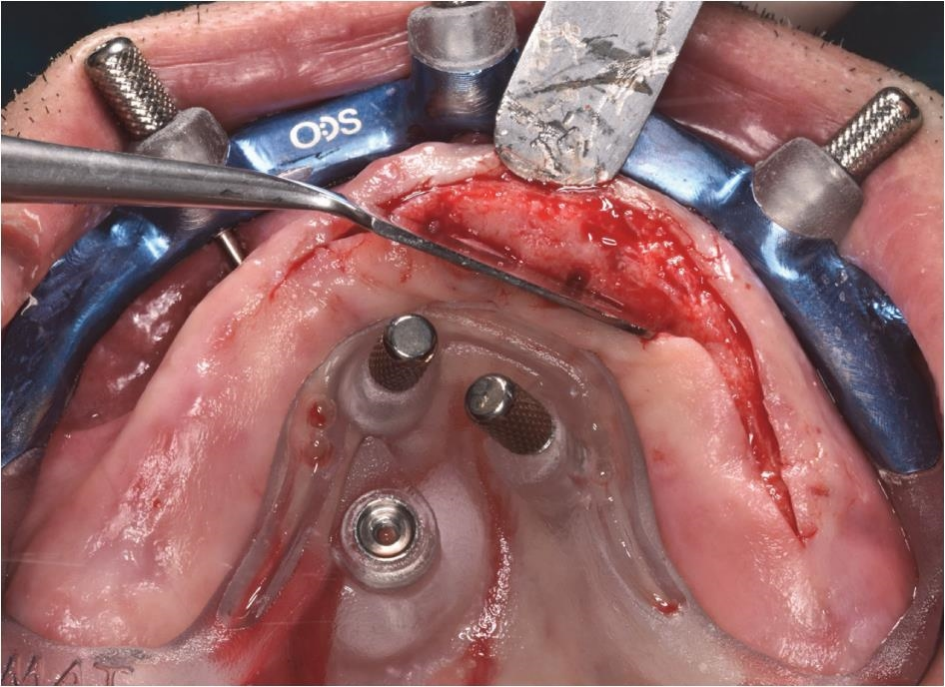
Surgical view after removal of the key template, with the base template remaining anchored in place. A limited crestal full‐thickness flap was performed from site 1.2 to 2.5 to minimize intraoperative bleeding and facilitate access for implant placement.

Under local anesthesia, the key template and base template were positioned intraorally and seated through occlusal contact, after which the base template was secured with bone fixation pins and the key template removed (Figure 4A–D). A limited crestal flap was elevated to expose the surgical field (Figure 5). The drive template was positioned to guide osteotomies and insertion of implants according to the planned three‐dimensional trajectories (Figure 6A–D).

Figure 4Extraoral and intraoral positioning of the key template screwed to the base template, showing occlusal and frontal views before intraoral transfer (A and B) and final intraoral seating confirming passive fit and correct occlusal alignment for subsequent guided surgical steps (C and D).

**Figure:**
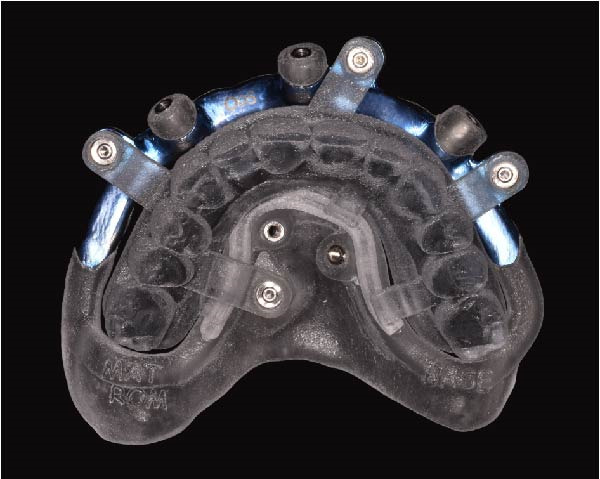
(A)

**Figure:**
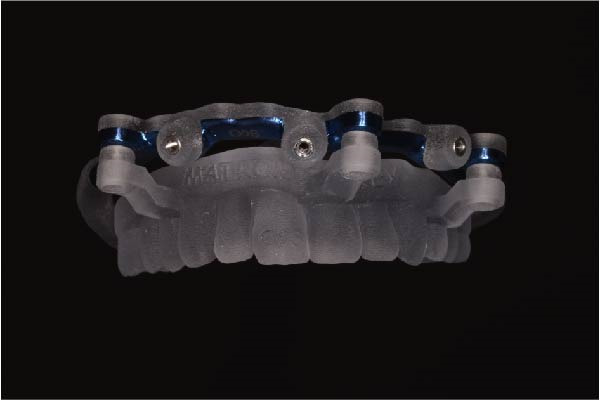
(B)

**Figure:**
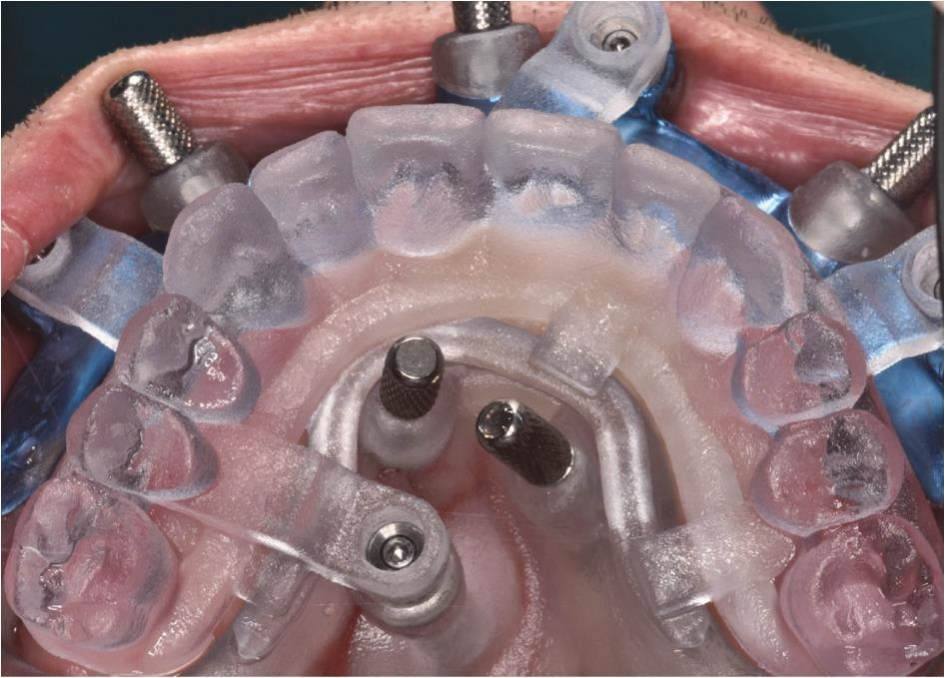
(C)

**Figure:**
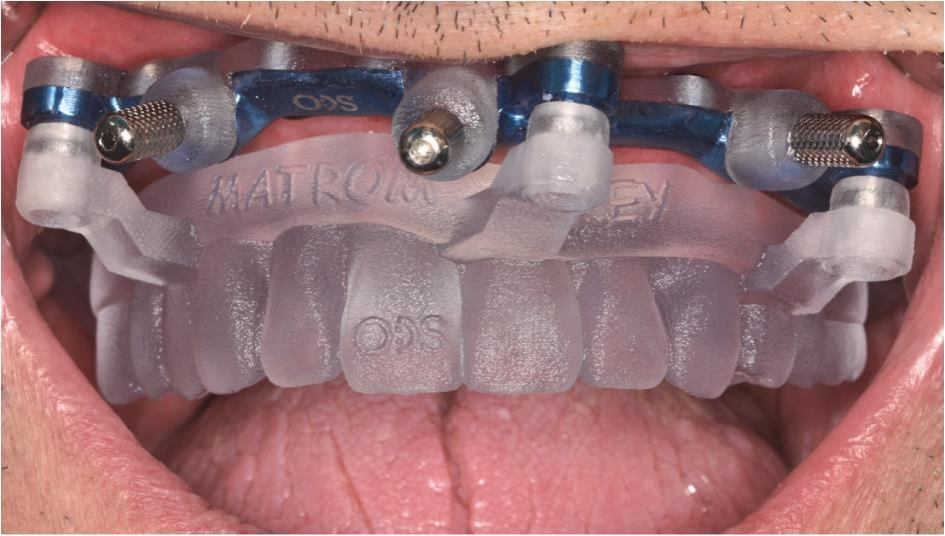
(D)

Figure 6Guided implant placement using the drive template screwed to the base template, showing extraoral occlusal and frontal views before intraoral transfer (A and B), followed by intraoral occlusal and frontal views confirming accurate guided insertion of implants in sites 1.2, 2.2, and 2.5 with proper angular positioning (C and D).

**Figure:**
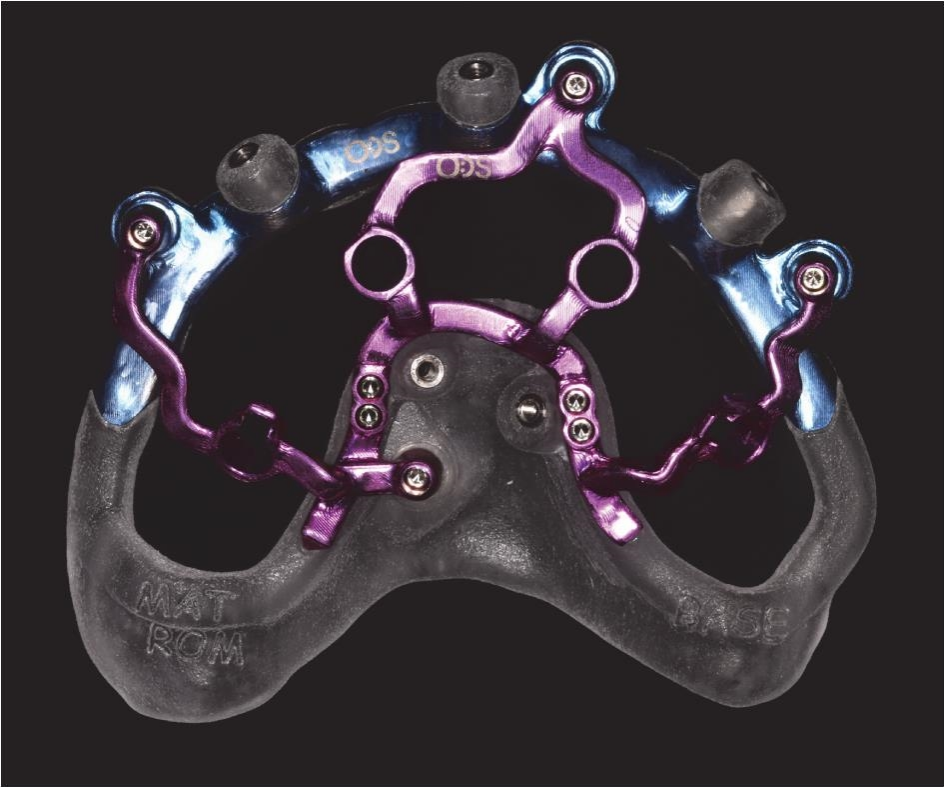
(A)

**Figure:**
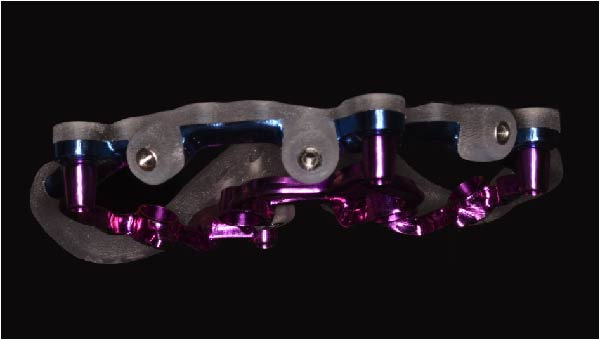
(B)

**Figure:**
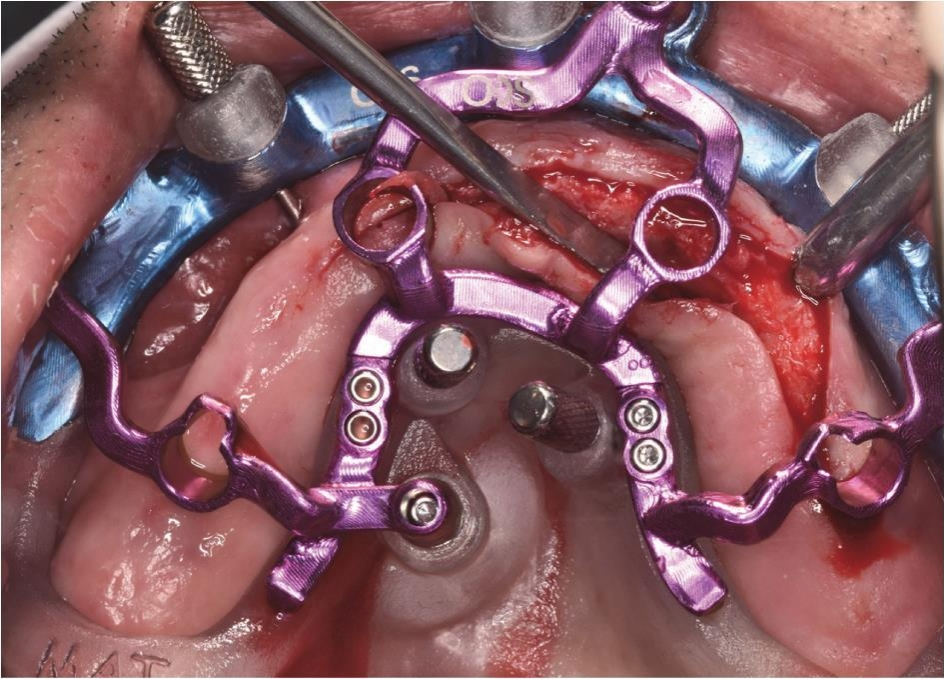
(C)

**Figure:**
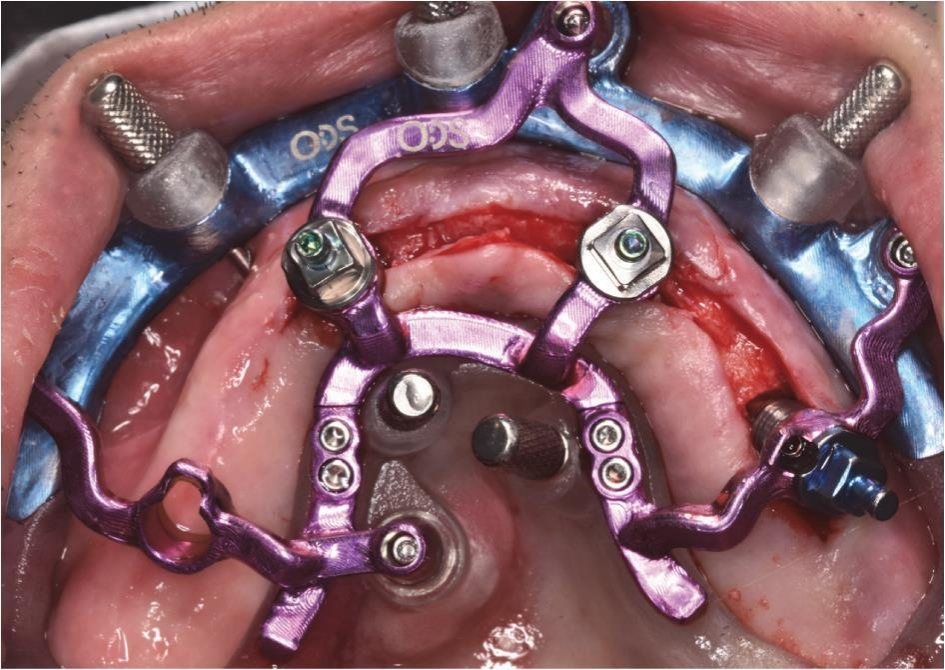
(D)

The antrostomy guide was then fixed onto the base template to outline the lateral window and allow guided elevation of the Schneiderian membrane for posterior trans‐sinus access (Figure 7A–E). The trans‐sinus implant (FIXO; Oxy Implant) was subsequently placed using the drive template to maintain planned angulation and depth, achieving an insertion torque of 50 Ncm, consistent with the torque values recorded for all implants placed in this procedure (Figure 8A, B). Height‐adjusted temporary abutments were connected, and the prosthetic template was used to facilitate relining and cementation of the provisional full‐arch prosthesis, performed extraorally prior to final seating (Figure 8C–H).

Figure 7Guided lateral sinus access using the antrostomy guide screwed to the base template, showing extraoral and intraoral positioning of the guide according to the virtual plan (A and B), initial osteotomy and creation of the lateral window along the predefined contours (C and D), and completion of Schneiderian membrane elevation preparing the site for trans‐sinus implant placement in 1.5 (E).

**Figure:**
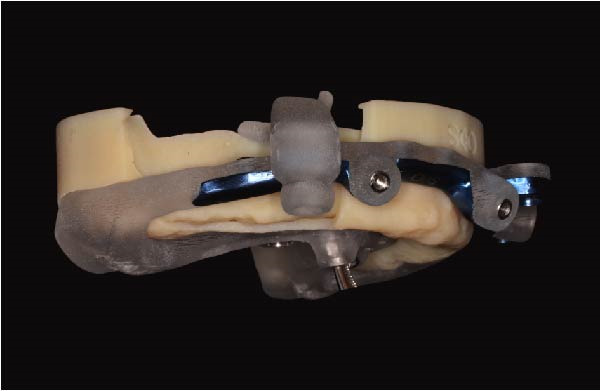
(A)

**Figure:**
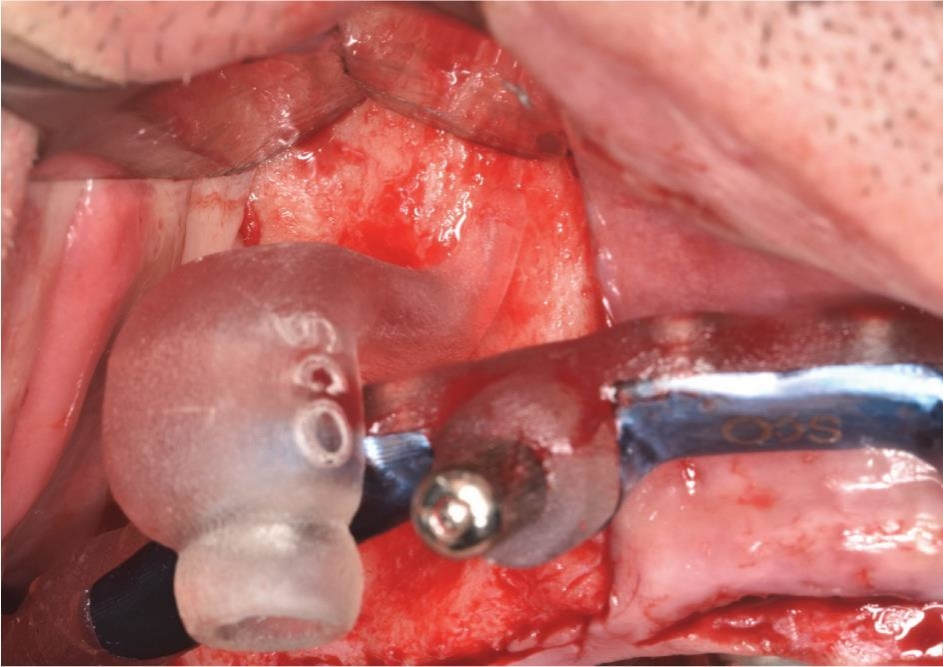
(B)

**Figure:**
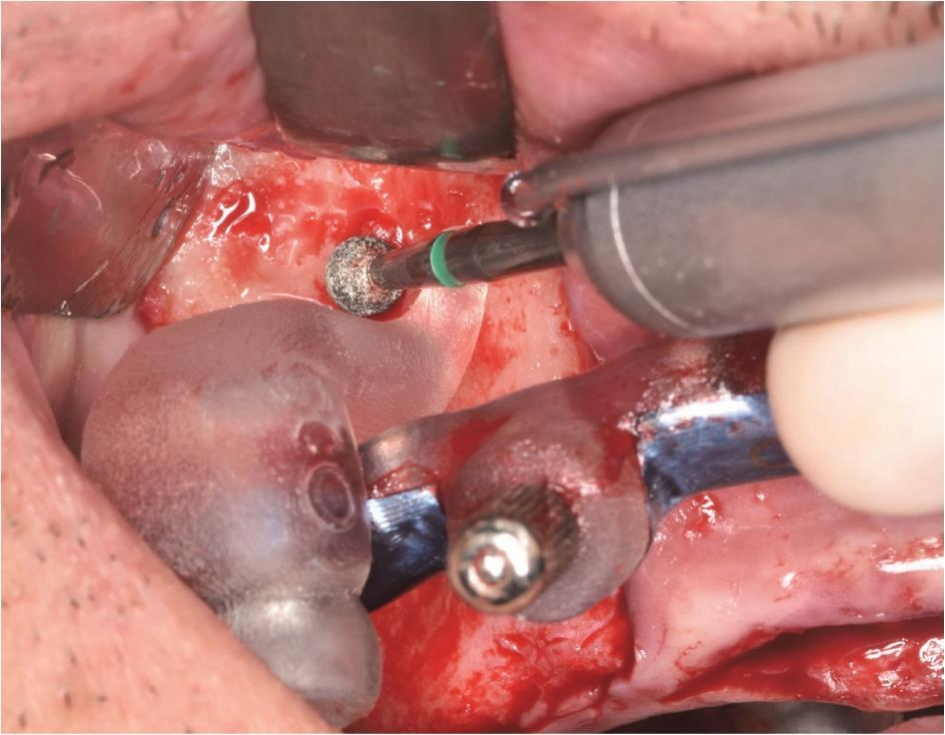
(C)

**Figure:**
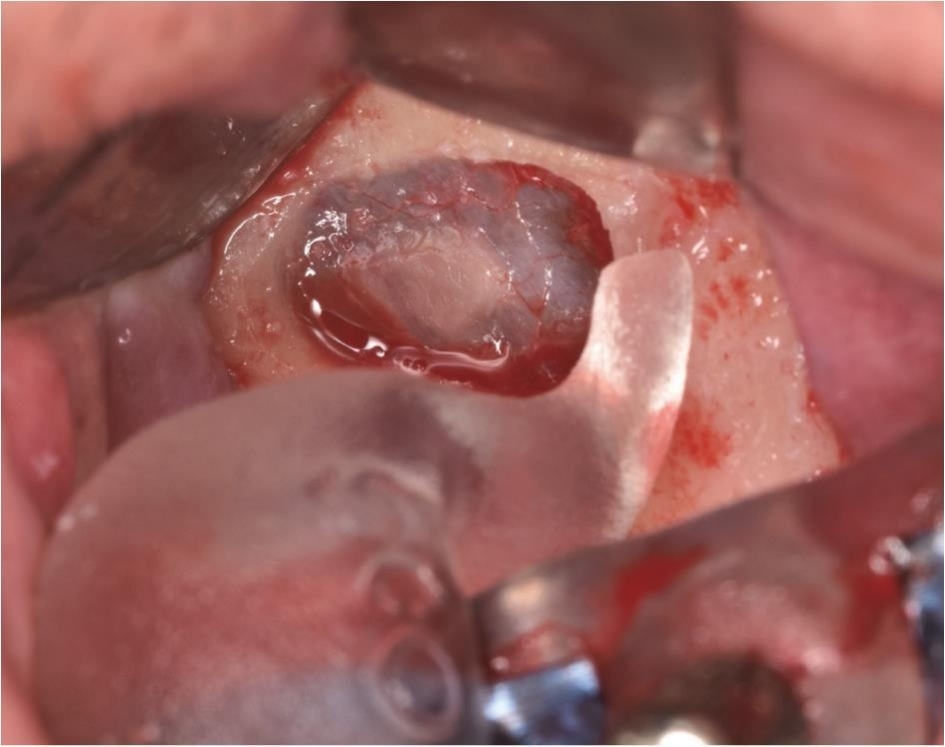
(D)

**Figure:**
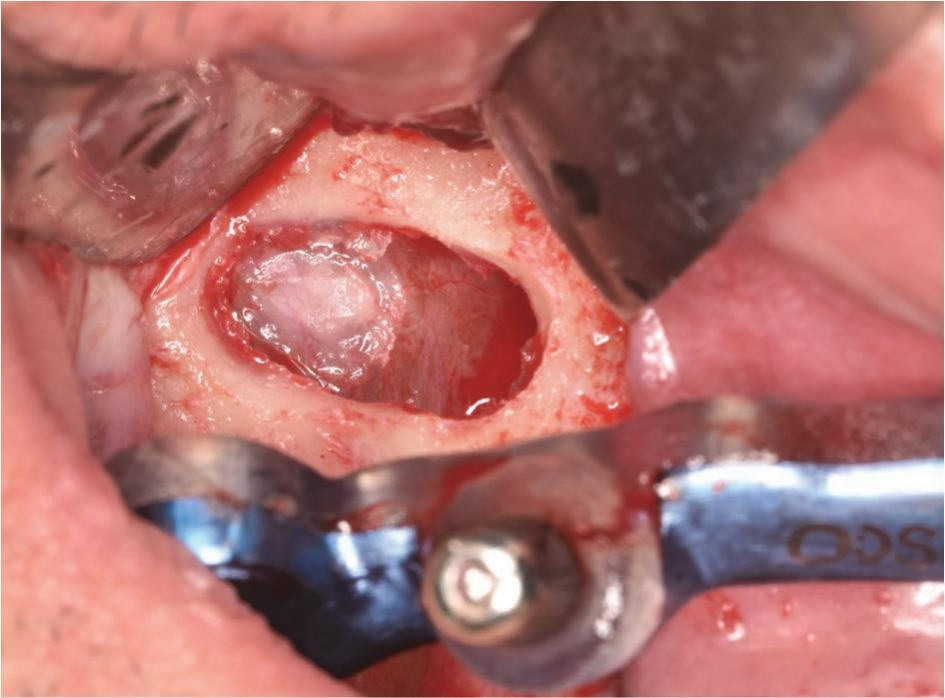
(E)

Figure 8Guided insertion of the trans‐sinus implant and prosthetic workflow, showing repositioning of the drive template after sinus membrane elevation to allow implant placement in site 1.5 (A and B), connection of height‐adjusted temporary abutments (C), seating of the prosthetic template for intraoral verification (D–F), and extraoral finishing and polishing of the PMMA provisional full‐arch prosthesis with metal reinforcement prior to delivery (G and H).

**Figure:**
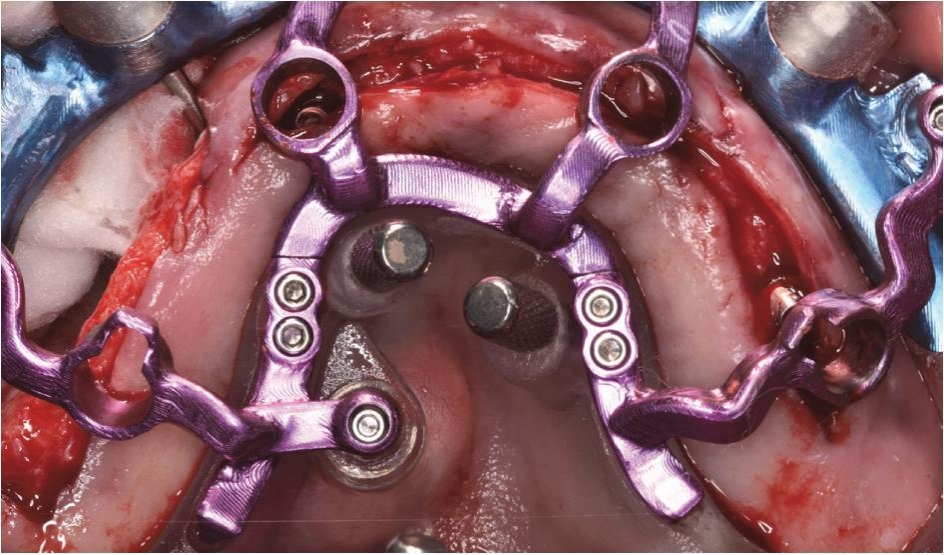
(A)

**Figure:**
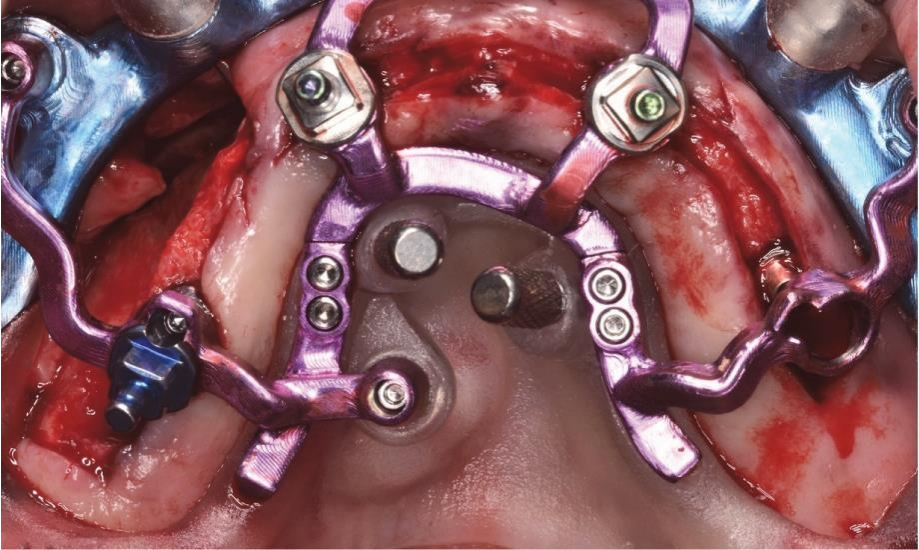
(B)

**Figure:**
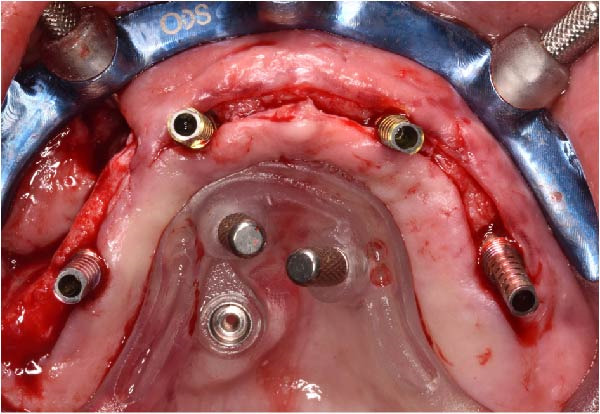
(C)

**Figure:**
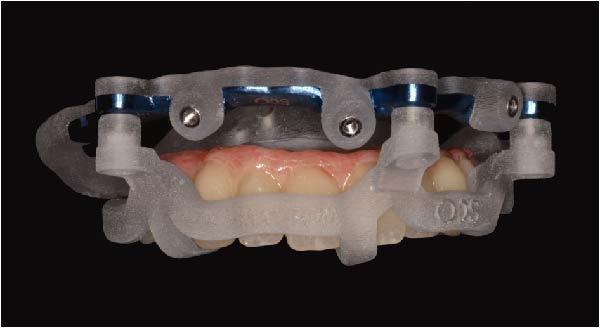
(D)

**Figure:**
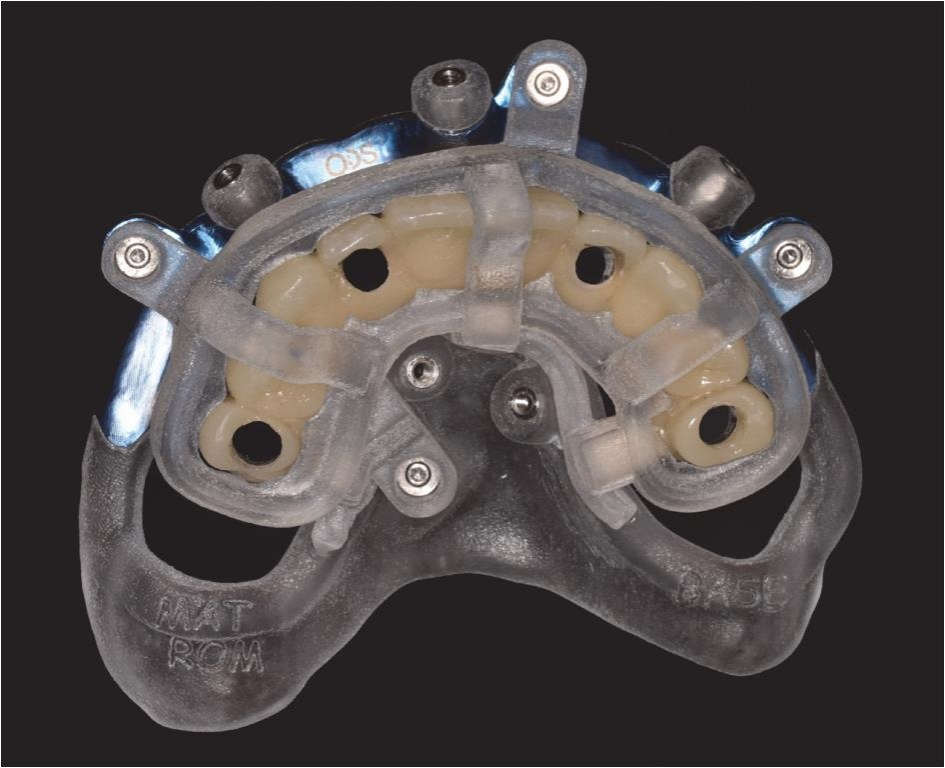
(E)

**Figure:**
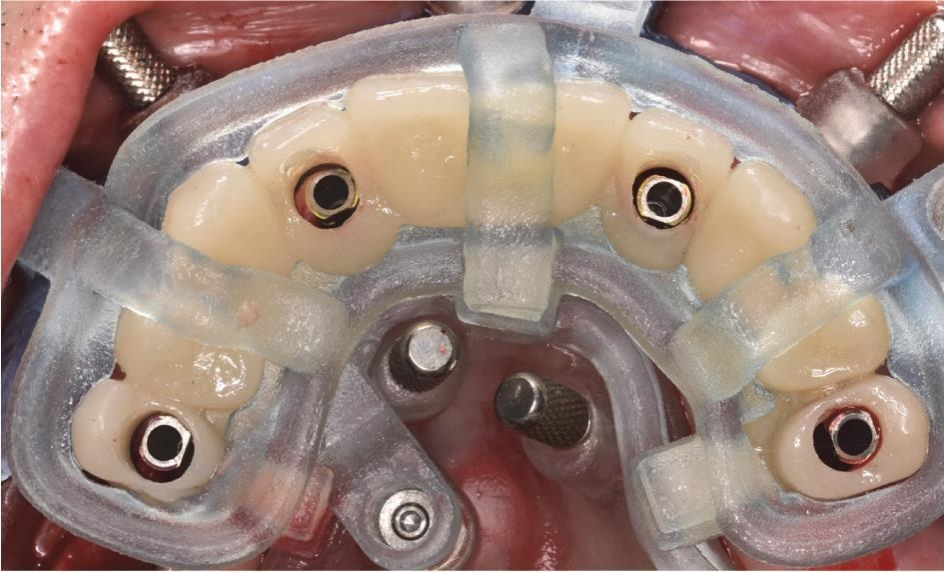
(F)

**Figure:**
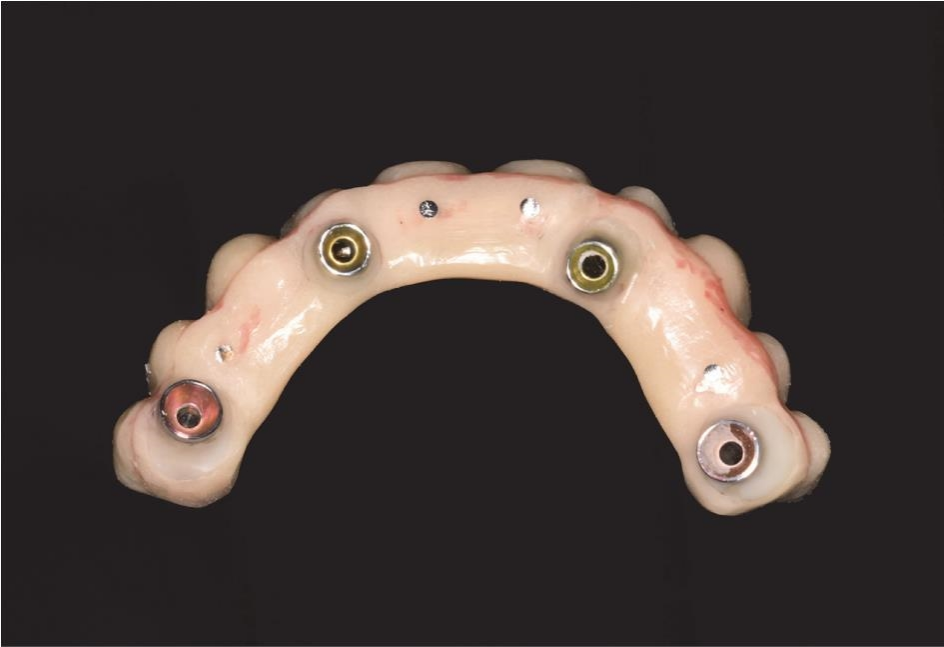
(G)

**Figure:**
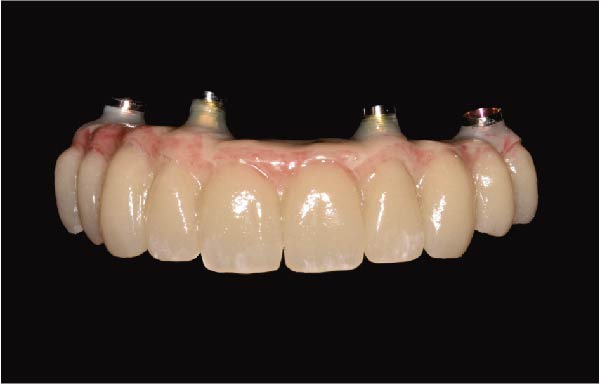
(H)

Upon completion, the flap was sutured, occlusion refined, and postoperative radiographs confirmed correspondence between planned and achieved implant positions (Figure 9A–D). The patient was discharged with postoperative instructions and scheduled for follow‐up evaluations. Table 1 shows the operative workflow for guided trans‐sinus full‐arch rehabilitation.

Figure 9Final surgical and prosthetic outcome showing flap closure with sutures and temporary healing abutments (A), occlusal and frontal views of the screw‐retained provisional full‐arch prosthesis after intraoral relining and occlusal adjustment (B and C), and immediate postoperative panoramic radiograph confirming final implant positioning (D).

**Figure:**
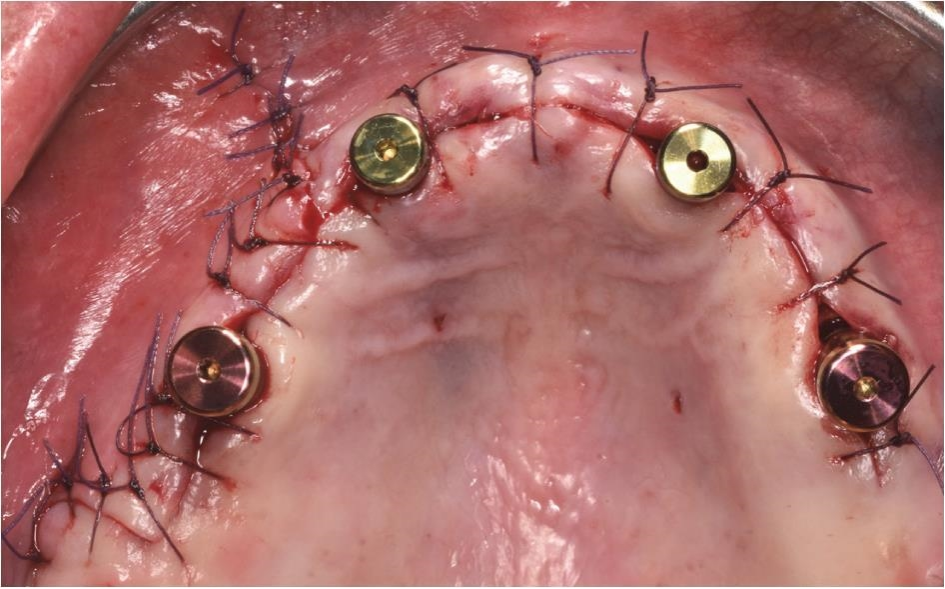
(A)

**Figure:**
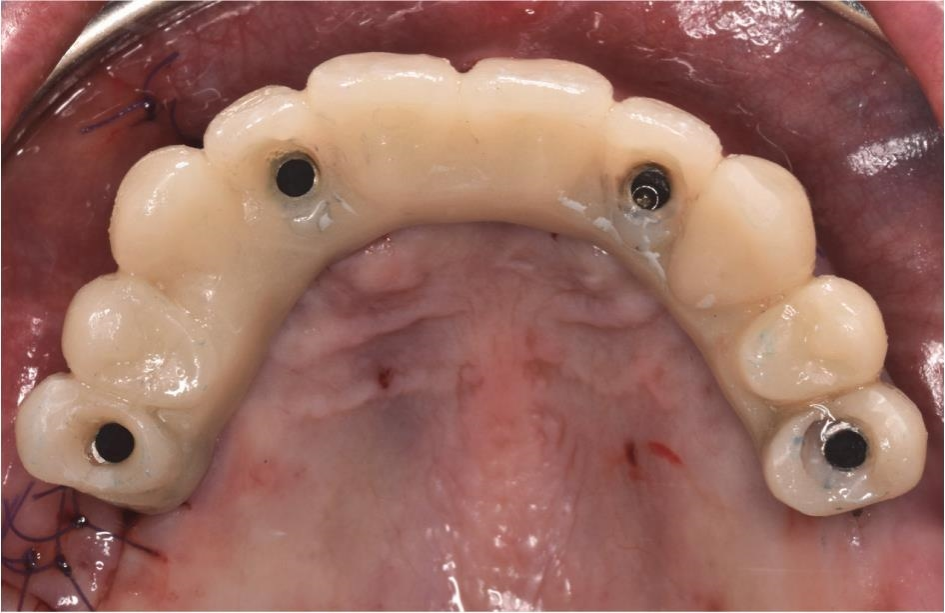
(B)

**Figure:**
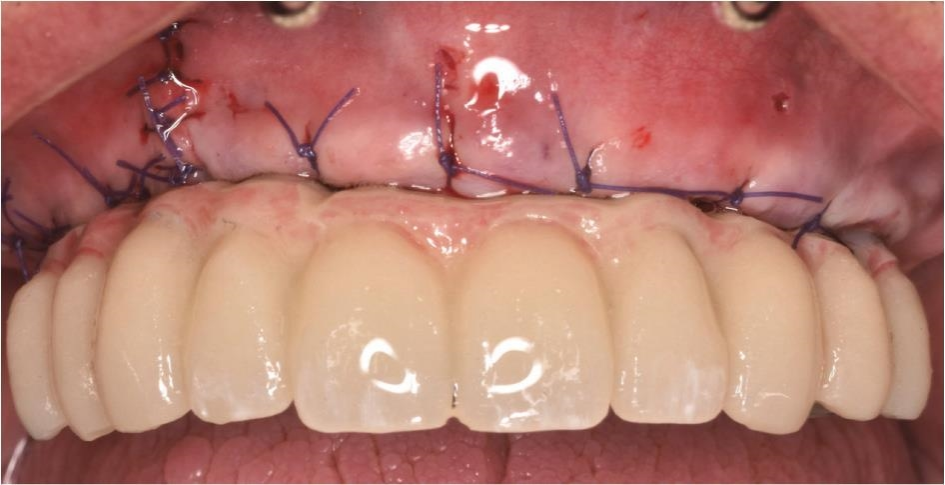
(C)

**Figure:**
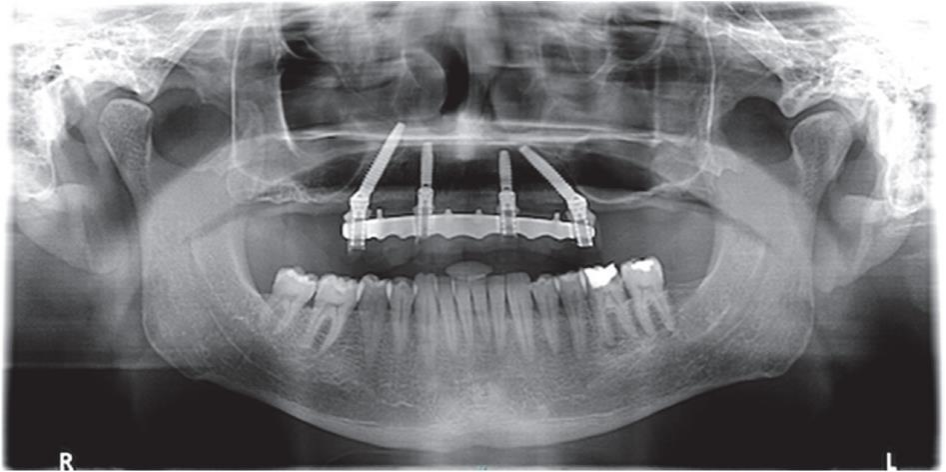
(D)

**Table 1:** Operative workflow for guided trans‐sinus full‐arch rehabilitation.

1. Clinical and radiographic assessment confirming posterior maxillary atrophy with sinus pneumatization and adequate anterior bone volume for implant anchorage.
2. Definition of treatment objectives: full‐arch implant‐supported rehabilitation using a guided trans‐sinus implant and stackable surgical system.
3. Obtaining informed consent from the patient prior to treatment initiation.
4. Taking preliminary maxillary impressions using alginate material (Hydrogum 5; Zhermack).
5. Taking definitive maxillary and mandibular impressions using polyvinyl siloxane (Elite HD+; Zhermack).
6. Recording maxillomandibular relationships and fabrication of complete removable denture for esthetic and functional reference.
7. Relining the denture with Soft‐Liner (GC Europe), followed by Soft Reline GC (GC Europe) after 2 months.
8. Scanning edentulous maxilla, prosthesis, opposing arch, and occlusion using TRIOS 5 (3Shape).
9. Importing STL files into CAD software for diagnostic virtual waxing (Exocad; exocad GmbH).
10. 3D printing diagnostic prototype with radiopaque markers using MED610 resin and J5 DentaJet (Stratasys).
11. Acquiring CBCT (GiANO HR; NewTom) and registering STL–DICOM data via RealGUIDE (3DIEMME).
12. Virtual implant planning, including tilted trans‐sinus implant to engage anterior sinus wall.
13. Designing stackable guide system (PCube; Oxy Implant Dental System): key template, base template, drive template, antrostomy guide, prosthetic template.
14. Positioning key template intraorally, seating through occlusion, and fixing base template with bone pins.
15. Removing key template and elevating crestal flap.
16. Positioning drive template and inserting implants fully guided according to planned trajectories (FIXO; Oxy Implant).
17. Positioning antrostomy guide and performing guided lateral window osteotomy.
18. Elevating Schneiderian membrane and placing trans‐sinus implant in site 1.5 under template guidance.
19. Connecting height‐adjusted temporary abutments according to virtual prosthetic plan.
20. Fixing prosthetic template and performing intraoral relining of provisional prosthesis.
21. Extraoral finishing and polishing of PMMA full‐arch provisional prosthesis with metal reinforcement.
22. Delivering screw‐retained provisional prosthesis with occlusal refinement and passive seating.
23. Suturing flap and verifying implant placement radiographically.
24. Two‐week follow‐up: suture removal, soft tissue evaluation, and hygiene reinforcement.

### 2.5. Treatment Results

At the 2‐week follow‐up appointment, clinical evaluation revealed satisfactory soft tissue healing around all implant sites, without signs of acute inflammation, dehiscence, or membrane exposure. The provisional full‐arch prosthesis demonstrated stable and passive seating, with no evidence of occlusal interferences or functional instability during mandibular movements (Figure 10A, B). All implants exhibited primary stability upon clinical assessment, and no postoperative complications such as sinus infection, neuropathic symptoms, or prosthetic decementation were reported. The patient reported good comfort during function and absence of significant pain or swelling following surgery. Customized hygiene instructions were reinforced to ensure proper maintenance of the provisional restoration and peri‐implant tissues. Scheduled follow‐up visits were planned to monitor osseointegration and soft tissue maturation prior to final prosthetic rehabilitation. At the 6‐month follow‐up, clinical examination confirmed the stability of the provisional full‐arch prosthesis and the maintenance of healthy peri‐implant soft tissues, with no signs of inflammation, mucosal recession, or prosthetic complications. The peri‐implant mucosa appeared well matured and well adapted around the transmucosal components. Frontal clinical view demonstrated satisfactory esthetic integration of the provisional restoration (Figure 11A), while the frontal view of the maxillary arch confirmed appropriate soft‐tissue contour and mucosal maturation (Figure 11B). The occlusal view of the screw‐retained provisional prosthesis confirmed stable seating on the implants (Figure 11C). The occlusal view of the healed implant sites with multiunit abutments in place showed healthy peri‐implant tissues without signs of inflammation (Figure 11D). Radiographic evaluation at 6 months showed stable implant positioning and absence of peri‐implant radiolucencies or sinus‐related complications (Figure 11E). The patient reported satisfactory masticatory function and continued comfort with the provisional restoration.

Figure 10A 2‐week follow‐up showing the provisional full‐arch prosthesis in place with sutures still present at the crestal and vertical incision sites (A), and postoperative clinical view after suture removal demonstrating satisfactory soft‐tissue healing around the implants (B).

**Figure:**
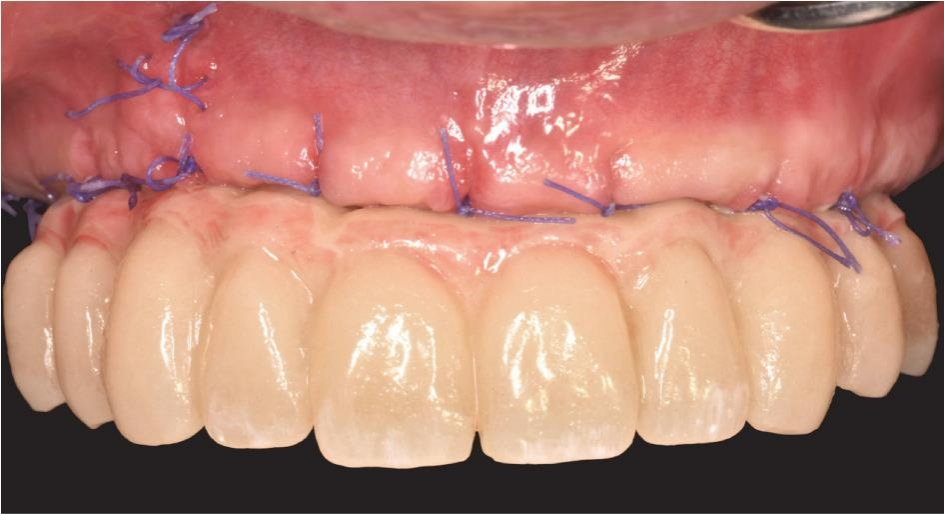
(A)

**Figure:**
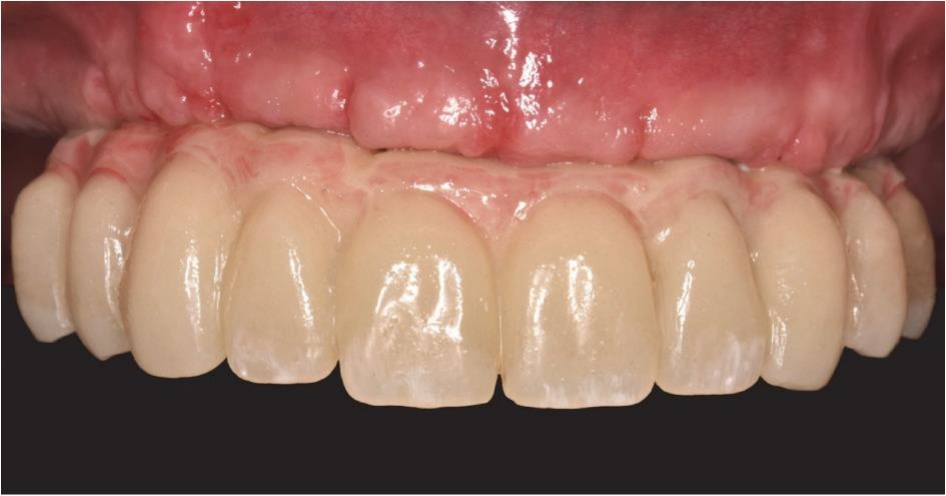
(B)

Figure 11A 6‐month follow‐up demonstrating the provisional full‐arch implant‐supported prosthesis and peri‐implant tissue conditions. Frontal clinical view showing stable esthetic integration of the provisional restoration (A). Frontal view of the maxillary arch highlighting satisfactory soft‐tissue contour and maturation of the peri‐implant mucosa (B). Occlusal view of the provisional screw‐retained prosthesis connected to the implants (C). Occlusal view of the implant sites after healing with the multiunit abutments in place, confirming healthy peri‐implant tissues (D). Panoramic radiograph at 6 months confirming stable implant positioning and absence of radiographic complications (E).

**Figure:**
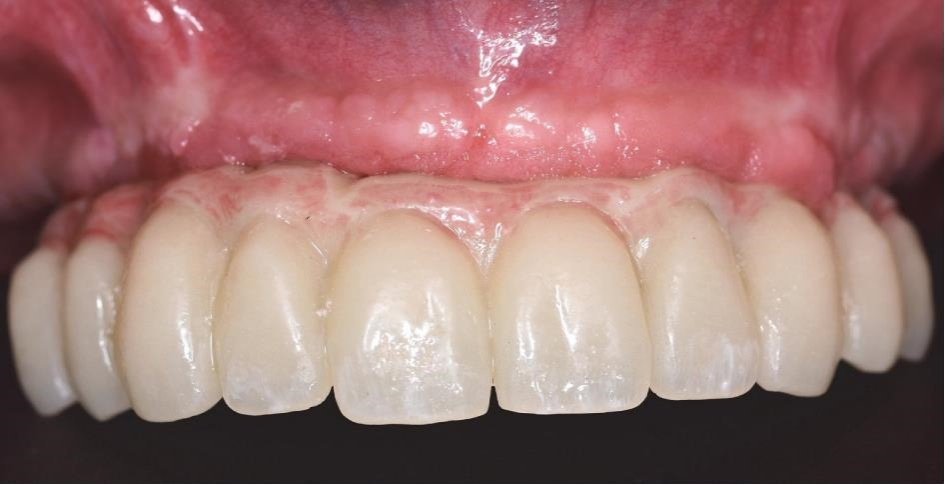
(A)

**Figure:**
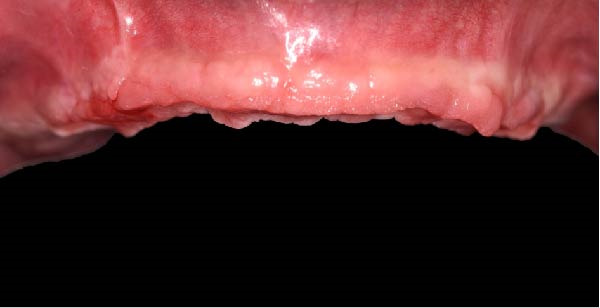
(B)

**Figure:**
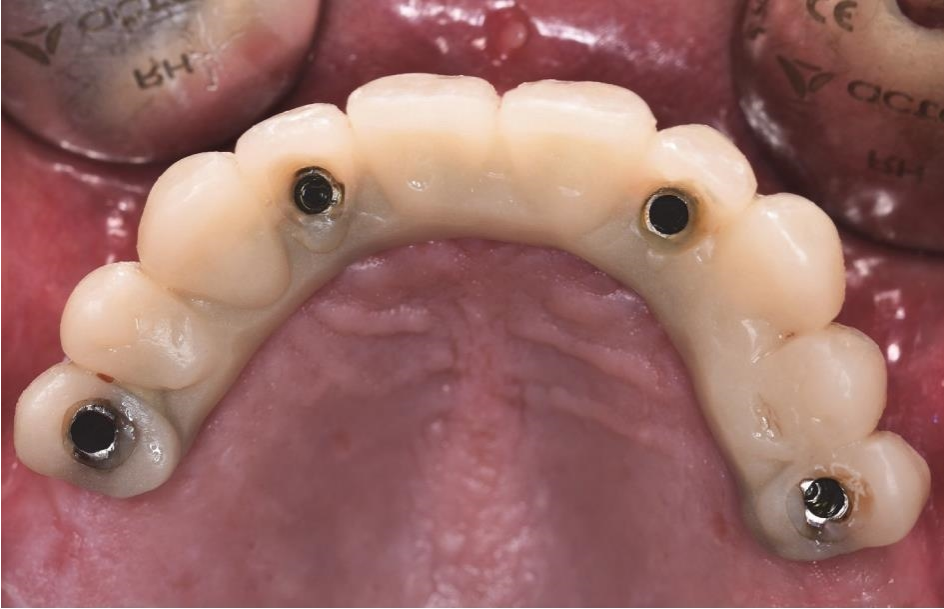
(C)

**Figure:**
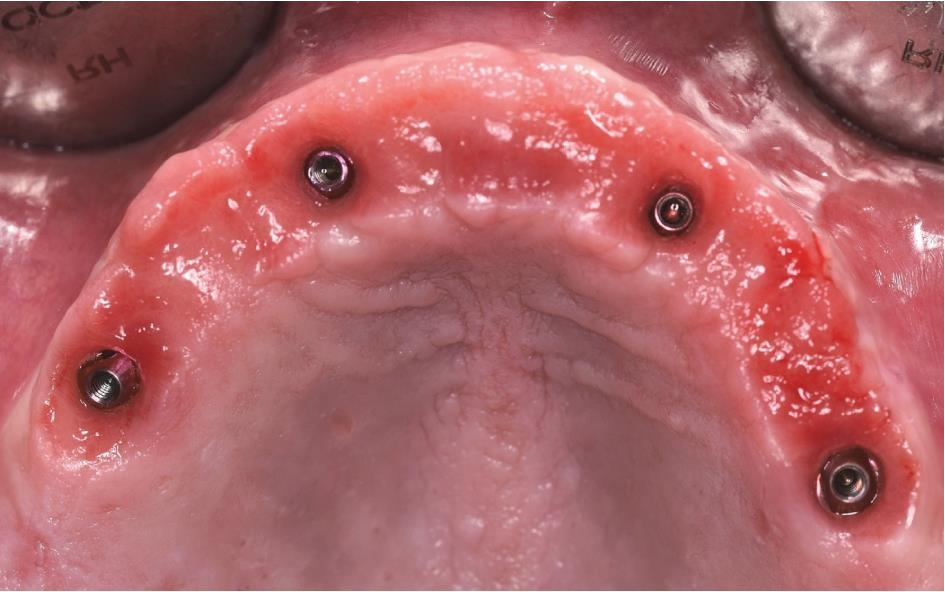
(D)

**Figure:**
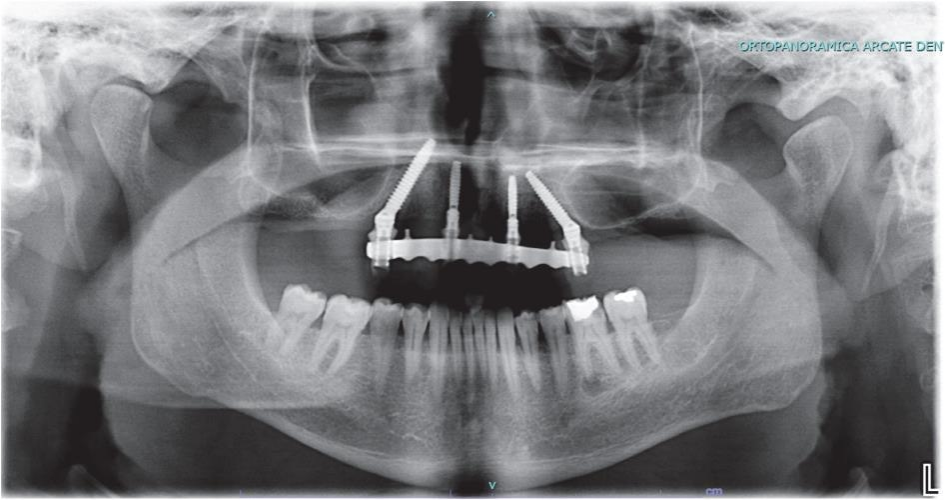
(E)

## 3. Discussion

The incorporation of a digitally planned antrostomy guide into a fully stackable guided surgery protocol introduces a new application of computer‐assisted implantology for full‐arch rehabilitation. Stackable templates have proven to be accurate, flexible, and reproducible, particularly in immediate‐loading scenarios in edentulous patients [1–6, 11]. Their modular design supports a stepwise workflow, facilitating bone reduction, guided implant placement, and prosthetic delivery with reduced surgical time and variability [2, 3, 5].

The addition of an antrostomy guide addresses the clinical need for controlled sinus access during trans‐sinus implant placement in atrophic posterior maxillae [12–17]. Accurate window positioning is essential to ensure safe entry, align with the planned implant trajectory, and avoid critical anatomical structures [14–16]. Traditional freehand techniques offer limited precision, and while static and dynamic navigation systems have been proposed for guided sinus lifts [21, 22], these are focused on augmentation procedures, not implant insertion through the sinus cavity.

No previous protocols have described the integration of a sinus window guide into a stackable workflow. This approach may improve surgical precision, minimize flap extension, and enable a seamless transition from sinus access to implant insertion, particularly in anatomically demanding cases requiring distant cortical anchorage [14–17].

From a radiological standpoint, the use of CBCT and postoperative imaging was carefully justified by the complexity of the case and the need for accurate three‐dimensional planning of the trans‐sinus implant trajectory. Although radiation exposure is always a relevant concern in implant dentistry, in the present scenario the diagnostic and surgical benefits of CBCT—particularly for evaluating sinus anatomy, residual bone height, cortical engagement, and the spatial relationship with critical anatomical structures—outweighed the potential risks. The imaging protocol was therefore selected according to the As Low As Reasonably Achievable (ALARA) principle [23], aiming to balance maximal surgical precision with minimal patient exposure.

Beyond full‐arch rehabilitation, the proposed protocol is applicable to segmental edentulism of the posterior maxilla. In these indications, the antrostomy guide enables controlled, reproducible lateral window creation that is coaxial with the prosthetically driven trans‐sinus implant trajectory. This alignment improves entry‐point precision, angulation, and depth control, thereby mitigating freehand‐related deviation and reducing interoperator variability. The advantage is particularly relevant in cases with limited RBH, narrow surgical corridors, or proximity to critical anatomical structures (e.g., alveolar antral artery, posterior superior alveolar vessels, sinus septa), where exact three‐dimensional implant positioning is required for predictable occlusal load distribution and long‐term prosthetic success [14–17].

Nevertheless, clinical application demands proper case selection and operator experience. Specific anatomical conditions—such as low sinus floors, fragile lateral walls, or altered sinus morphology—may limit suitability. In addition, the cost and technical complexity associated with fabricating and using multiple guide components should be weighed against the potential clinical benefits [6, 11].

Although the present report includes a 6‐month clinical and radiographic follow‐up demonstrating stable implant positioning, satisfactory peri‐implant tissue healing, and absence of sinus‐related complications, the findings should be interpreted with caution. As a single case report, this study primarily demonstrates the feasibility of the proposed guided antrostomy protocol and its integration within a stackable workflow, rather than establishing long‐term clinical predictability. Further clinical studies with larger patient cohorts and extended follow‐up periods are required to confirm the long‐term reliability, prosthetic performance, and biological stability of this approach.

## 4. Conclusions

Within the limitations of this case report, integrating a digitally planned antrostomy guide into a stackable surgical system appears to allow precise sinus access and trans‐sinus implant placement within a fully guided full‐arch workflow. The technique enabled accurate translation of the virtual plan, supported immediate loading, and showed uneventful early healing with stable conditions at 6‐month follow‐up, suggesting its potential usefulness in managing complex posterior maxillary rehabilitations. Further studies with larger samples and longer follow‐up are required to confirm the long‐term reliability of this approach.

**Nomenclature**
ALARA: As Low As Reasonably Achievable
CAD: Computer‐aided design
CBCT: Cone‐beam computed tomography
DICOM: Digital imaging and communications in medicine
PMMA: Polymethyl methacrylate
RBH: Residual bone height
STL: Standard triangulation language.

## Author Contributions

**Mattia Manfredini**: conceptualization, data curation, investigation, methodology, project administration, resources, software, supervision, validation, visualization, writing – review and editing. **Matteo Pellegrini**: validation, visualization, writing – original draft. **Pier Paolo Poli**: validation, visualization, writing – review and editing. **Carlo Maiorana**: project administration, supervision, validation, visualization. **Franco Sanseverino**: methodology, resources, software, validation, visualization. **Mario Beretta:** conceptualization, data curation, investigation, methodology, project administration, resources, software, supervision, validation, visualization, writing – review and editing.

## Funding

This case report did not get any external funding. Open access publishing facilitated by Universita degli Studi di Milano, as part of the Wiley ‐ CRUI‐CARE agreement.

## Consent

Written informed consent was obtained prior to the report.

## Conflicts of Interest

The authors declare no conflicts of interest.

## Data Availability

The data that support the findings of this study are available from the corresponding author upon reasonable request.
